# Does FGF21 Mediate the Potential Decrease in Sweet Food Intake and Preference Following Bariatric Surgery?

**DOI:** 10.3390/nu13113840

**Published:** 2021-10-28

**Authors:** Mette S. Nielsen, Christian Ritz, Anne Chenchar, Wender L. P. Bredie, Matthew P. Gillum, Anders Sjödin

**Affiliations:** 1 Novo Nordisk Foundation Center for Basic Metabolic Research, Faculty of Health and Medical Sciences, University of Copenhagen, 2200 Copenhagen, Denmark; mette.snielsen@sund.ku.dk (M.S.N.); achencha@uwyo.edu (A.C.); gillum@sund.ku.dk (M.P.G.); 2 National Institute of Public Health, University of Southern Denmark, 1455 Copenhagen, Denmark; ritz@sdu.dk; 3 School of Pharmacy, College of Health Science, University of Wyoming, Laramie, WY 82071, USA; 4 Department of Food Science, Faculty of Science, University of Copenhagen, 1958 Frederiksberg, Denmark; wb@food.ku.dk; 5 Department of Nutrition, Exercise and Sports, Faculty of Science, University of Copenhagen, 1958 Frederiksberg, Denmark

**Keywords:** fibroblast growth factor 21, roux-en-Y gastric bypass, sleeve gastrectomy, food preference, taste preference, sweet taste sensitivity

## Abstract

The liver-derived hormone fibroblast growth factor 21 (FGF21) has recently been linked to preference for sweet-tasting food. We hypothesized, that surgery-induced changes in FGF21 could mediate the reduction in sweet food intake and preference following bariatric surgery. Forty participants (35 females) with severe obesity (BMI ≥ 35 kg/m^2^) scheduled for roux-en-y gastric bypass (*n* = 30) or sleeve gastrectomy (*n* = 10) were included. Pre- and postprandial responses of intact plasma FGF21 as well as intake of sweet-tasting food assessed at a buffet meal test, the hedonic evaluation of sweet taste assessed using an apple juice with added sucrose and visual analog scales, and sweet taste sensitivity were assessed before and 6 months after bariatric surgery. In a cross-sectional analysis pre-surgery, pre- and postprandial intact FGF21 levels were negatively associated with the hedonic evaluation of a high-sucrose juice sample (*p* = 0.03 and *p* = 0.02). However, no changes in pre- (*p* = 0.24) or postprandial intact FGF21 levels were found 6 months after surgery (*p* = 0.11), and individual pre- to postoperative changes in pre- and postprandial intact FGF21 levels were not found to be associated with changes in intake of sweet foods, the hedonic evaluation of sweet taste or sweet taste sensitivity (all *p* ≥ 0.10). In conclusion, we were not able to show an effect of bariatric surgery on circulating FGF21, and individual postoperative changes in FGF21 were not found to mediate an effect of surgery on sweet food intake and preference.

## 1. Introduction

Changes in food and taste preferences away from calorie-dense high-fat and sweet foods are considered a key contributor to the decline in calorie intake after bariatric surgery—the main driver for postoperative weight loss. Following surgery, patients report decreased craving and interest for sweets [1,2,3], decreased sweet taste palatability when tasting sucrose [1,2,4], and decreased self-reported intake of sweets (e.g., candy, cake, chocolate) [5,6,7], although inconsistency across studies does exist [8].

A growing body of evidence suggests altered hormone responses as mediators implicated in changes in food preferences and taste function following bariatric surgery. GLP-1 receptor knockout mice display reduced responsiveness to sweet stimuli [9,10], and GLP-1 and PYY are expressed in mammalian taste buds and their respective receptors are found on intragemmal taste afferent nerve fibers [9,11,12,13], suggesting a role for these hormones in modulating taste perception.

The liver-derived hormone fibroblast growth factor 21 (FGF21) has recently been linked to sweet preference in both pre-clinical and clinical studies. In rodents, administration of FGF21 selectively suppresses consumption of sucrose and non-caloric sweeteners [14,15], whereas FGF21-knockout mice increase consumption of a high-sucrose diet and intake of liquid sucrose, glucose, and fructose solution during two-bottle preference tests [15]. Furthermore, administration of a long-acting FGF21 analog significantly reduces intake of saccharin in non-human primates [14]. In humans, genetic variation in the FGF21 gene is linked to increased sweet consumption. Søberg et al. showed that carriers of the rs838133 A-allele of FGF21 had higher self-reported candy consumption and were ~20% more likely to consume large amounts of sweet food. Furthermore, plasma FGF21 increased after sucrose consumption, and fasting FGF21 was ~50% higher in those that do not enjoy sweet foods than in those that do [16]. More recently, large genome-wide association studies have confirmed the link between FGF21 and sugar intake in humans [17,18]. Based on these findings, we speculate, that altered FGF21 responses following bariatric surgery could be a mediator implicated in changes in sweet food intake and preference after surgery.

The effect of bariatric surgery on FGF21 is still unclear. Circulating FGF21 levels seem to increase in the early postoperative period, whereas unchanged or decreased levels are seen after the first postoperative year [19,20,21,22,23,24,25]. A recently published review and meta-analysis concluded that changes in FGF21 after bariatric surgery are affected by follow-up duration, with increased FGF21 initially after Roux-en-Y gastric bypass (RYGB) but decreased levels after the first postoperative year [26]. Little is known about a potential effect of altered FGF21 levels after bariatric surgery, yet it seems unlikely that FGF21-signalling play an important role for the postoperative weight loss [27]. However, it may still have a potential contributing role in changes in taste and food preferences sometimes reported after bariatric surgery.

To our knowledge, no studies have investigated if surgery-induced changes in FGF21 could mediate an effect of surgery on sweet food intake and preference. This study, therefore, aimed to investigate if (1) FGF21 is associated with sweet food and taste preference in patients with severe obesity scheduled for bariatric surgery, (2) bariatric surgery leads to changes in FGF21 and (3) if altered FGF21 levels are associated with changes in sweet food and taste preferences following bariatric surgery.

## 2. Materials and Methods

### 2.1. Participants

Forty patients scheduled for RYGB or sleeve gastrectomy (SG) at Køge Hospital, Denmark, were included in the analyses. Participants were recruited between March 2014 and July 2015. Eligibility for bariatric surgery in Denmark included age ≥ 25 year and BMI ≥ 50 kg/m^2^ or ≥35 kg/m^2^ with complications of obesity. Furthermore, national guidelines required a weight loss of ≥8% of initial body weight before surgery. Exclusion criteria for the present study included pregnancy as well as inability, physically or mentally, to comply with the procedures required by the study protocol. All participants gave written informed consent.

### 2.2. Design

This study was part of a larger study that aimed to identify the multiple factors determining the variation in weight loss after bariatric surgery [28]. A full description of the study design has been published previously [29].

Pre- and postprandial FGF21, as well as a number of tests evaluating sweet food intake, hedonic evaluation of sweet taste, and sweet taste sensitivity, were assessed before bariatric surgery and again 6 months after surgery. On each test day, participants arrived at the Department of Nutrition, Exercise and Sports, University of Copenhagen, at 9 a.m. (after 12 h fast). Anthropometric data were collected, and a standardized liquid meal (Cambridge Weight Plan^®^, 954 kJ) was served at 10 a.m. From 11 a.m. to 12 noon, sensitivity and hedonic rating tests were carried out at the sensory laboratory of the adjacent Department of Food Science, University of Copenhagen. A standardized liquid test meal (Cambridge Weight Plan^®^, 1674 kJ, 120 g whole milk, 100 g semi-skimmed milk, and 80 g powder, 39% carbohydrate, 36% protein, and 25% fat) was served at 1.15 p.m. Blood samples were drawn before the meal (this served as the preprandial blood sample but was not a true fasting sample) and 6 times postprandial (15, 30, 60, 90, 120, and 180 min). Furthermore, liking for sweets was assessed from visual analog scales (VAS) before the meal and 6 times postprandial. A buffet meal test assessing food preferences was served at 4.30 p.m. (the buffet meal test was served on a test day 3 months before surgery and not on the test day 1–2 weeks before surgery).

The study was approved by the Scientific Ethics Committees of the Capital Region of Denmark (J.no H-32013138) and registered in the database www.clinicaltrials.gov (accessed on 12 August 2021) (ID no NCT02070081).

### 2.3. Outcomes

Anthropometrics: Body weight and height were measured in the morning after an overnight fast. Body weight was measured to the nearest 0.1 kg and height was measured to the nearest 0.5 cm using a wall-mounted digital stadiometer.

Biochemical measures: Blood samples were analyzed for intact (i.e., bioactive) FGF21 1–2 weeks before surgery and 6 months after surgery. Blood was drawn into chilled EDTA tubes. Samples were immediately cooled on ice and centrifuged at 4 °C. All blood samples were frozen at −80 °C until analyzed. Samples were analyzed for concentrations of intact FGF21 taking into account the recently described proteolytic cleavage of FGF21 by dipeptidyl peptidase IV and fibroblast activation protein [30,31]. FGF21 was analyzed using N- and C-terminally directed antibodies to detect full-length, active protein according to the manufacturer’s instructions (F2131, EagleBiosciences, Nashua, NH, USA).

Sweet food intake: The following 20 food items with high or low fat content, combined with high or low sugar content were served at the buffet meal 3 months before surgery (and before the mandatory 8% diet-induced weight-loss period) and 6 months after surgery: pork rib roast, chicken fillet strips, nuggets, fish cakes, Danish omelet, French fries, creamy potato gratin, bread, mayonnaise, ketchup, remoulade, skyr with berries, cut raw vegetables, cut fruit, vanilla ice cream, chocolate sauce, cocoa meringues, biscuit cones with chocolate, sweet licorice, and Danish pastries. The only liquid served at the meal was water. Participants ate unaccompanied and were instructed to eat according to their preferences for as long as they wanted. The amount consumed (g) and energy intake (kJ) were registered. The participants were unaware of this registration [32]. Only total carbohydrate intake and intake from the sweet category (i.e., biscuit cones with chocolate, chocolate sauce, cocoa meringues, vanilla ice cream, sweet licorice, skyr with berries, Danish pastries, ketchup, cut fruit, and remoulade) were used in the analysis presented here.

Hedonic rating of sweet: Hedonic rating of sweet was assessed 1–2 weeks before surgery and 6 months after surgery and was assessed using an apple juice (10 °C, Organic, Rynkeby, Denmark) with 5, 10, 15, and 20 g/L added sucrose (only the sample with 20 g/L sucrose was used in the analysis presented here). All samples were presented in 30-mL cups, and the participants consumed water (200 mL) and crackers (5 g) to rinse their palate. Each sample was rated on a Danish translation of the 9-point (−4 to 4) hedonic scale [33]. Furthermore, VAS (10 cm) before the test meal, and 15, 30, 60, 90, 120, and 180 min after the meal estimated liking for sweet. The scale ranged from 0 cm (“I do not want to eat something sweet”) to 10 cm (“I do want to eat something sweet”).

Sensitivity for sweet taste: Detection threshold (i.e., the minimum value of the taste stimulus needed to give rise to a sensation) and recognition threshold (i.e., minimum value of the taste stimulus permitting identification of the sensation perceived) for sweet taste was carried out according to ISO 3972 [34], which is based on the ascending method of limits, 1–2 weeks before surgery and 6 months after surgery. Nine aqueous solutions with increasing concentration of sucrose (99.5%, Sigma-Aldrich, USA; 0 g/L, 0.34 g/L, 0.55 g/L, 0.94 g/L, 1.56 g/L, 2.59 g/L, 4.32 g/L, 7.2 g/L, 12 g/L) were presented to the participants. Detection and recognition thresholds for each participant and each visit were determined as a best-estimated threshold (BET). The BET is defined as the geometric mean of the concentration at which the participant is estimated to report the correct detection or recognition threshold [35].

### 2.4. Statistical Analysis

There is no sample size calculation available for the present study, which is secondary analyses of data from a study where the primary endpoint was weight loss [28]. Thus, the statistical analyses carried out in this manuscript were not part of the pre-registered study plan and should be seen as exploratory.

The area under the curve (AUC) was calculated as total area using the trapezoidal rule. Participant characteristics were summarized as mean ± standard deviation (SD). All associations were analyzed using linear regressions. The preoperative association between FGF21 and sweet food and taste preference was performed as a cross-sectional analysis before surgery (i.e., in patients with obesity scheduled for bariatric surgery). Repeated measurements (changes in FGF21 across time) were analyzed using linear mixed models with a visit-time interaction, with within-visit participant-specific random effects, and with FGF21 logarithmically transformed to obtain normal distribution. In case a significant visit-time interaction was found, pairwise comparisons for each time point during the meal test were carried out using post hoc, model-based approximate t-tests. All models were adjusted for age and gender. Data are reported as regression coefficients (β) ± standard error of mean (SEM) and R^2^ where appropriate.

*p*-values less than 0.05 were considered significant. No adjustment for multiple testing was applied. Statistical analyses were carried out using R [36] and RStudio version 1.1.383 (www.rstudio.com) (accessed on 12 August 2021).

## 3. Results

Forty participants (35 females) with a mean age of 40.1 ± 9.4 years and a preoperative (1–2 weeks before surgery) BMI of 42.0 ± 6.2 kg/m^2^ were included in the analysis. Thirty participants received RYGB surgery and 10 received SG. Preoperative BMI and age did not differ between RYGB and SG participants (both *p* ≥ 0.22). Ten participants were diagnosed with type 2 diabetes before surgery.

### 3.1. Weight Loss after Bariatric Surgery

Mean weight loss 6 months after bariatric surgery was 25.0 ± 1.3 kg (*p* < 0.01) and BMI decreased with 8.7 ± 0.5 kg/m^2^ (*p* < 0.01). Weight loss did not differ between RYGB and SG participants (26.2 ± 1.5 kg versus 21.5 ± 2.6 kg, *p* = 0.12), but weight loss was larger in participants without type 2 diabetes compared to participants with type 2 diabetes (26.6 ± 1.5 kg versus 20.1 ± 2.5 kg, *p* = 0.03).

### 3.2. Associations between FGF21 and Sweet Food Intake, Hedonic Evaluation of Sweet Taste, and Sweet Taste Sensitivity in Patients with Obesity Scheduled for Bariatric Surgery

In the cross-sectional analysis before surgery, pre- and postprandial intact FGF21 levels were negatively associated with the hedonic evaluation of an apple juice with 20 g sucrose—with higher intact FGF21 levels being associated with lower hedonic rating of the highly sweet juice sample (*p* = 0.03 and *p* = 0.02). Furthermore, preprandial intact FGF21 tended to be negatively associated with recognition threshold for sweet taste—with higher intact FGF21 being associated with lower recognition threshold for sweet taste (*p* = 0.08). No pre-surgery associations were found between pre- and postprandial intact FGF21 and intake of sweet foods, total carbohydrate intake, or detection threshold for sweet taste (all *p* ≥ 0.08) (Table 1).

### 3.3. Effect of Bariatric Surgery on Circulating Intact FGF21

**Figure 1:**
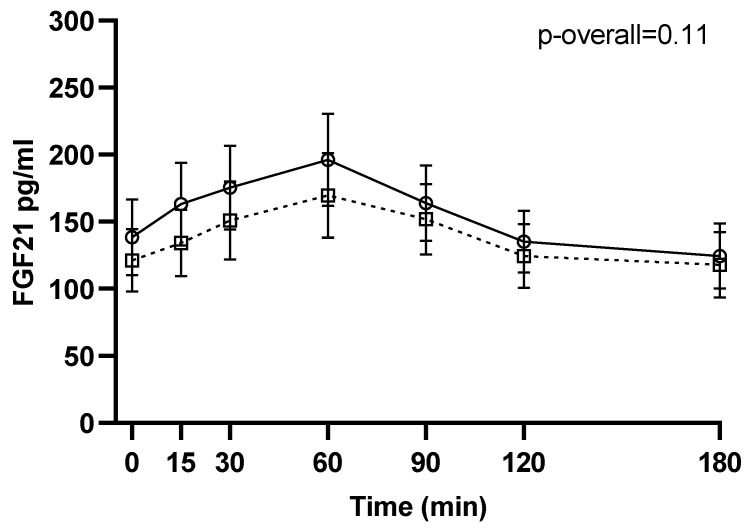
FGF21 during a three-hour mixed meal test approximately 1–2 weeks before (□, black dotted line) and 6 months after (○, black filled line) surgery. Raw data shown as mean ± SEM. *p*-overall was obtained from a repeated measurements linear mixed model including a visit-time interaction and age and gender as fixed effects and participant as random effect. FGF21: fibroblast growth factor 21.

**Figure 2:**
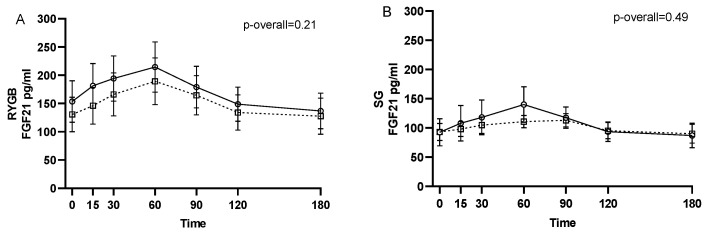
FGF21 during a three-hour mixed meal test approximately 1–2 weeks before (□, black dotted line) and 6 months after (○, black filled line) surgery in roux-en-y gastric bypass (RYGB) participants (**A**) and sleeve gastrectomy (SG) participants (**B**). Raw data shown as mean ± SEM. *p*-overall was obtained from a repeated measurements linear mixed model including a visit-time interaction and age and gender as fixed effects and participant as random effect. FGF21: fibroblast growth factor 21.

No changes from 1–2 weeks before surgery to 6 months after surgery were found for neither pre- (*p* = 0.24) nor postprandial intact FGF21 levels (*p* = 0.11 for the visit-time interaction) (Figure 1). Likewise, when stratifying by type of surgery, no changes in intact FGF21 were found from 1–2 weeks before surgery to 6 months after RYGB or SG (*p* = 0.21 and *p* = 0.49, respectively, for the visit-time interaction) (Figure 2).

### 3.4. Associations between Pre- to Postoperative Changes in FGF21 and Changes in Sweet Food Intake, Hedonic Evaluation of Sweet Taste, and Sweet Taste Sensitivity

Individual pre- to postoperative changes in pre- and postprandial intact FGF21 levels were not found to be associated with changes in intake of sweet foods, total carbohydrate intake, the hedonic evaluation of sweet or sweet taste sensitivity (all *p* ≥ 0.10) (Table 2).

When looking at associations in RYGB patients only, still no associations were found between changes in pre- and postprandial intact FGF21 levels and changes in intake of sweet foods, total carbohydrate intake, the hedonic evaluation of sweet or sweet taste sensitivity (all *p* ≥ 0.10).

## 4. Discussion

Although our data do not provide evidence for an effect of bariatric surgery on FGF21 six months after surgery or a mediating role of FGF21 in changes in sweet food and taste preferences, it adds to the existing literature by showing that FGF21 is linked to sweet preference in humans with severe obesity.

Pre-clinical and human genetic studies have identified FGF21 as a regulator of intake of sweet-tasting food [14,15,16,17,18]. Our data support a link between FGF21 and sweet preference in humans, including people with severe obesity. In a cross-sectional analysis in patients scheduled for bariatric surgery, we showed that participants with higher FGF21 levels had lower hedonic ratings for a highly sweet juice sample and tended to be more responsive to recognizing sweet taste. The regulatory effect of FGF21 on sweet preference is suggested to occur through a negative feedback loop between the liver and the brain [37]. Intake of simple sugars induces transcription of FGF21 in the liver and thereby increased circulating FGF21, through activation of the transcription factor ChREBP. FGF21 then activates neurons in the hypothalamus leading to a decreased preference for and intake of sweet-tasting foods [37]. However, to fully understand the regulatory role of FGF21 in humans, there is a need for human studies investigating the effect of FGF21 administration on sweet preference and potential mechanisms involved.

So far, no consensus has been reached as to whether bariatric surgery leads to changes in FGF21. The majority of studies investigating changes in FGF21 following surgery report increased pre- and postprandial FGF21 levels in the early postoperative period (i.e., within 3 months) after RYGB [19,21,22,23]. FGF21 seems to return to pre-surgery levels after the first postoperative year [25,38,39], indicating that FGF21 only transiently increases after surgery. We were not able to show any changes in FGF21 six months after RYGB and SG, or after RYGB when stratifying by type of surgery. Thus, the effect of surgery on FGF21 may already level off after the first postoperative months. However, there seem to be large interindividual variations in FGF21 [40], also in response to bariatric surgery [19,23]. In our sample changes in pre- and postprandial (AUC) FGF21 ranged from −213 to +403 pg/mL and −46,077 to +51,259 pg/mL*min, respectively. The significance of this variation in response to surgery is not known.

We hypothesized, that the individual variation in FGF21 in response to surgery could mediate an effect of surgery on sweet food intake and preference seen in some patients. We were, however, not able to confirm this hypothesis, since individual changes in FGF21 were not associated with changes in intake of sweet foods or the hedonic evaluation of sweet taste in our cohort. Data from the buffet meal test and the test assessing the hedonic evaluation of sweet taste used in present study have previously been reported [32,41,42]. On the group level, we found no pre- to postoperative changes in the hedonic evaluation of sweet taste or intake of sweet food from the buffet meal. However, changes in energy density of the buffet meal were associated with the postoperative weight loss indicating that some patients improve food choice after surgery with implications for the weight loss [42]. Studies assessing changes in intake of sweet-tasting foods such as cake, candy and, chocolate in the early postoperative period and at multiple time points afterward tend to report that the effect wanes with time after surgery [5,6,43,44]. Thus, it could be speculated if transient increases in FGF21 could be linked to transient reductions in intake of sweet-tasting foods within the first postoperative months.

The strengths of the current study were the measurement of bioactive FGF21 as well as the inclusion of multiple tests assessing sweet taste sensitivity and preference for sweet-tasting foods, including the buffet meal test assessing actual eating behavior. Limitations of the present study include the observational design based on associations and not causal relationships, the risk of false-positive findings given the few significant associations, and the fact that the sample size is biased towards women. The link between FGF21 and preference for sweet taste shown in the present study, therefore, needs to be confirmed in larger observational studies with more participants, and equal gender distribution and causality need to be verified in randomized controlled trials. Another limitation is the selection of sweet food items served at the buffet meal test. The buffet meal test was designed to assess general changes in food preferences after bariatric surgery [32], and the 20 food items served at the meal were organized into the following categories, high-fat sweet, low-fat sweet, high-fat savory, and low-fat savory. In the present analysis, we combined the categories high-fat sweet and low-fat sweet to assess if FGF21 was associated with sweet food intake. Besides cakes and candy, these categories also included ketchup, remoulade, fruit, and skyr with berries. The inclusion of these food items may have affected our results. Finally, sweet food intake at the buffet meal was assessed 2–3 months before surgery and before the 8% diet-induced weight-loss period whereas FGF21 was assessed 1–2 weeks before surgery. The fact that these two endpoints were assessed at two different time points, and when the participants were in different metabolic states, could have affected our results.

## 5. Conclusions

In conclusion, bariatric surgery did not lead to any significant changes in FGF21 six months after surgery, and we were not able to detect an association between individual changes in FGF21 and changes in intake of sweet foods, the hedonic evaluation of sweet taste, or sweet taste sensitivity. Thus, our data do not provide evidence for a mediating role of FGF21 in the potential changes in sweet food and taste preferences after bariatric surgery. However, our data may indicate, that a link between FGF21 and preference for sweet taste exists in humans with severe obesity. This link needs to be confirmed in further studies, especially in randomized controlled trials where FGF21 is pharmacologically elevated.

## Figures and Tables

**Table 1:** Cross-sectional associations 1–2 weeks before bariatric surgery between FGF21 and measures of sweet preference and intake and sweet taste sensitivity.

	β ± SEM	R^2^	*p*-Value
**Preprandial FGF21**			
Subjective appetite for sweet, preprandial	0.002 ± 0.003	24.5	0.42
Subjective appetite for sweet, AUC	0.27 ± 0.41	13.8	0.52
Hedonic rating of an apple juice with 20 g sucrose	−0.005 ± 0.002	15.9	0.03
Intake of carbohydrate (kJ) ^1^	0.04 ± 0.86	11.9	0.97
Intake of food from the sweet category (%) ^1^	0.02 ± 0.01	13.1	0.12
Detection threshold (g/L)	−0.002 ± 0.003	7.5	0.52
Recognition threshold (g/L)	−0.01 ± 0.01	16.7	0.08
**Postprandial FGF21 (AUC)**			
Subjective appetite for sweet, AUC	0.002 ± 0.002	14.7	0.37
Hedonic rating of an apple juice with 20 g sucrose	−0.00001 ± 0.00001	17.6	0.02
Intake of carbohydrate (kJ) ^1^	−0.0003 ± 0.004	11.7	0.95
Intake of food from the sweet category (%) ^1^	0.0001 ± 0.0001	9.6	0.26
Detection threshold (g/L)	−0.00001 ± 0.0001	7.6	0.49
Recognition threshold (g/L)	−0.00005 ± 0.00003	14.0	0.13

^1^ Assessed 3 months before surgery and before an 8% weight loss. The remaining variables were assessed 1–2 weeks before surgery. Associations were evaluated using linear regression models adjusted for age and gender. AUC, area under the curve; FGF21: fibroblast growth factor 21.

**Table 2:** Associations between changes in FGF21 and changes in sweet preference and intake and sweet taste sensitivity from before surgery to 6 months after Roux-en-Y gastric bypass and sleeve gastrectomy.

	β ± SEM	R^2^	*p*-Value
**ΔPreprandial FGF21**			
ΔSubjective appetite for sweet, preprandial	0.01 ± 0.01	11.2	0.27
ΔSubjective appetite for sweet, AUC	−0.45 ± 0.70	5.4	0.53
ΔHedonic rating of an apple juice with 20 g sucrose	0.004 ± 0.004	3.7	0.29
ΔIntake of carbohydrate (kJ) ^1^	0.13 ± 1.21	9.2	0.91
ΔIntake of food from the sweet category (%) ^1^	−0.05 ± 0.03	11.9	0.10
ΔDetection threshold (g/L)	−0.003 ± 0.01	14.8	0.65
ΔRecognition threshold (g/L)	0.003 ± 0.01	2.6	0.73
**ΔPostprandial FGF21 (AUC)**			
ΔSubjective appetite for sweet, AUC	0.0002 ± 0.004	4.3	0.95
ΔHedonic rating of an apple juice with 20 g sucrose	0.00002 ± 0.00002	3.7	0.41
ΔIntake of carbohydrate (kJ) ^1^	0.002 ± 0.01	10.0	0.81
ΔIntake of food from the sweet category (%) ^1^	−0.0003 ± 0.0002	12.1	0.13
ΔDetection threshold (g/L)	−0.00002 ± 0.00003	11.9	0.50
ΔRecognition threshold (g/L)	0.00002 ± 0.00004	3.4	0.61

^1^ Assessed 3 months before surgery and before an 8% weight loss. The remaining variables were assessed 1–2 weeks before surgery. Associations were evaluated using linear regression models adjusted for age and gender. AUC, area under the curve; FGF21: fibroblast growth factor 21.

## Data Availability

The datasets generated during and/or analyzed during the current study are not publicly available but are available from the corresponding author on reasonable request.
